# Complete genome sequencing of *Lacticaseibacillus rhamnosus* NS2301G1 isolated from kimchi

**DOI:** 10.1128/mra.00141-25

**Published:** 2025-08-11

**Authors:** Kiyeop Kim, ByungWook Son, JongHoon Kim, Sejong Oh

**Affiliations:** 1 Division of Animal Science, Chonnam National University 34931, Gwangju, Republic of Korea; 2 Research & Development Center, Nong Shim Co. Ltd. 372214 , Seoul, Republic of Korea; University of Maryland School of Medicine, Baltimore, Maryland, USA

**Keywords:** *Lacticaseibacillus rhamnosus *NS2301G1, complete genome sequence, probiotic, kimchi

## Abstract

Sequencing of the *Lacticaseibacillus rhamnosus* NS2301G1 strain isolated from kimchi revealed a 2,968,519 bp circular chromosome with a 46.74% GC content; in addition, genes linked to probiotic properties, stress tolerance, and carbohydrate metabolism were identified, highlighting the strain’s potential application in the biotechnology and food industries.

## ANNOUNCEMENT

**Fig 1:**
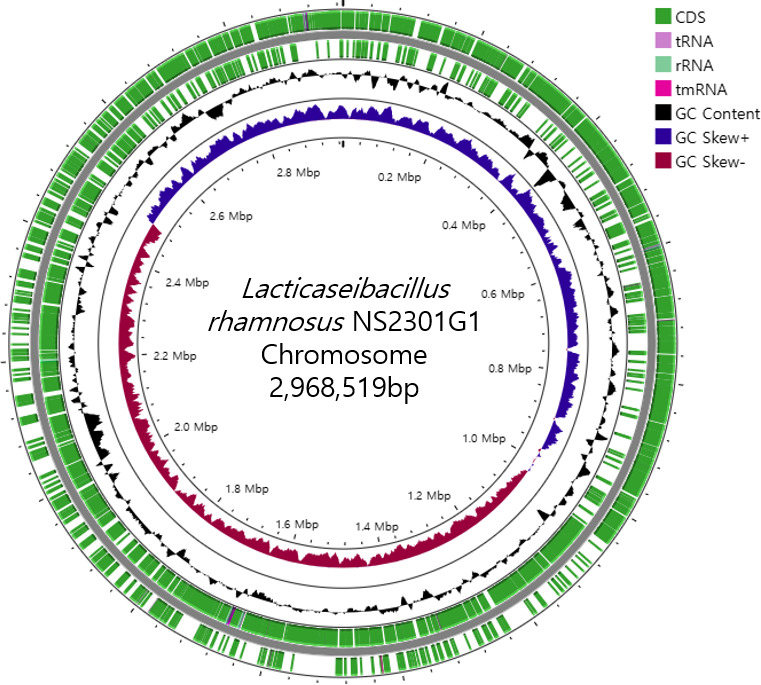
Circular chromosome map of *L. rhamnosus* NS2301G1. Marked features are shown from the periphery to the center: protein coding sequences on the forward strand, protein coding sequences on the reverse strand, tRNA, rRNA, GC ratio, and GC skew. bp, base pair; G, guanine; C, cytosine; tRNA, transfer RNA; rRNA, ribosomal RNA.

Lactic acid bacteria (LAB) are widely recognized for their roles in fermentation and potential as probiotics (1). *Lacticaseibacillus rhamnosus* NS2301G1 was isolated from kimchi collected in Gwangju, Republic of Korea. To isolate this strain, kimchi was homogenized in sterile saline and spread on de Man–Rogosa–Sharpe (MRS) agar (pH 5.0), followed by anaerobic incubation at 37°C for 48–72 h (2). Colonies with typical LAB morphology were screened by catalase and Gram staining tests. Taxonomic identity was confirmed by 16S rRNA gene sequencing using primers 27F and 1492R and Sanger sequencing, followed by BLASTn analysis. For genome analysis, NS2301G1 was cultured in MRS broth (pH 6.8) at 37°C for 24 h under anaerobic conditions. Genomic DNA was extracted using the DNeasy Blood & Tissue Kit (Qiagen). Two sequencing libraries were prepared for Illumina and Oxford Nanopore Technologies (ONT) platforms. Illumina libraries were prepared with an average insert size of ~450 bp. Illumina sequencing generated 2.45 million clean paired-end reads, while ONT yielded 103,939 long reads after filtering. No DNA shearing or size selection was performed for ONT. The N50 values were 12,310 bp before and 12,400 bp after filtering. Illumina reads were trimmed using Trimmomatic v0.39 with the parameters LEADING:10, TRAILING:10, SLIDINGWINDOW:4:20, and MINLEN:200 (3). Reads mapping to the phiX genome were removed using Bowtie2 v2.3.5.1 with default settings, followed by filtering with SAMtools v1.9. Nanopore reads were basecalled using Guppy v3.1.5 and filtered using NanoFilt v2.8.0 to exclude reads with a mean Phred quality score <7 and length <1,000 bp (4–6). Hybrid *de novo* assembly was performed using Unicycler v0.4.8 with default settings (7). The final assembly resulted in one circular chromosome and four plasmids (Fig. 1). Genome annotation was performed using the NCBI Prokaryotic Genome Annotation Pipeline (PGAP, 2023-11-20.build6894) (8). According to PGAP, the genome contains 2,755 protein-coding genes, 61 pseudogenes, 61 tRNAs, and 15 rRNAs (Table 1). The difference in gene counts is attributed to variations in pseudogene prediction and ORF filtering algorithms. The genome topology (circular vs linear) was inferred computationally based on contig end continuity, without experimental validation. The sequences were not rotated to a specific origin or gene during assembly. Functional annotation identified genes associated with probiotic activity (e.g., *dltA*, *luxS*, and *srtA*), stress tolerance (*clpB*, *dnaK*), and oxidative stress response (*trxA_1*, *trxA_2*). These features suggest that *L. rhamnosus* NS2301G1 may possess enhanced survivability and colonization capacity under gastrointestinal and oxidative conditions. This complete genome sequence enhances our understanding of the genetic basis underlying probiotic functionality in LAB and provides a valuable resource for further industrial and functional food applications.

**TABLE 1:** Summary of PGAP-based genome features of *L. rhamnosus* NS2301G1^*a*^^,^^*b*^

Statistics	CP166134.1	CP166135.1	CP166136.1	CP166137.1	CP166138.1
Genome length (bp)	2,968,519	64,513	45,896	11,830	4,076
GC content (%)	46.74	43.50	42.07	40.77	43.55
Illumina depth	373.861	655.628	345.558	454.864	175.707
Nanopore depth	279.41	424.827	202.335	214.009	68.4899
CDS	2,816	72	46	11	5
Protein-coding	2,755	52	41	9	5
Pseudogene	61	20	5	2	0
tRNA	61	0	0	0	0
rRNA	15	0	0	0	0

^*a*^ CDS, protein-coding genes, and pseudogenes were annotated using PGAP. All tRNAs and rRNAs were identified only on the chromosome (CP166134.1, https://www.ncbi.nlm.nih.gov/nuccore/CP166134.1/). Contigs 2 to 5 represent plasmids (CP166135.1, https://www.ncbi.nlm.nih.gov/nuccore/CP166135.1/ to CP166138.1, https://www.ncbi.nlm.nih.gov/nuccore/CP166138.1/).

^*b*^ bp, base pair; G, guanine; C, cytosine; CDS, coding sequence; tRNA, transfer RNA; rRNA, ribosomal RNA.

## Data Availability

The complete genome sequence has been deposited in GenBank under the accession numbers CP166134.1 (chromosome) and CP166135.1 to CP166138.1 (plasmids). The BioProject accession number is PRJNA1141725, and the BioSample accession number is SAMN43023992. The raw sequencing reads have been deposited in the NCBI Sequence Read Archive (SRA) under the accession numbers SRR33365613 (Illumina) and SRR33365612 (Nanopore). The 16S rRNA gene sequence has been deposited in GenBank under the accession number PV717307.
